# The Well-Being of Doctoral Students in Education: An Ecological Systems Perspective

**DOI:** 10.3390/bs14100929

**Published:** 2024-10-10

**Authors:** Wendan Xu, Yingxiu Li, Ronnel B. King, Junjun Chen

**Affiliations:** 1 Department of Education Policy and Leadership, Education University of Hong Kong, Hong Kong SAR, China; s1130087@s.eduhk.hk (W.X.); s1129964@s.eduhk.hk (Y.L.); 2 Department of Curriculum and Instruction, Faculty of Education, The Chinese University of Hong Kong, Hong Kong SAR, China; rbking@cuhk.edu.hk

**Keywords:** doctoral students, well-being, influential factors, ecological systems theory, interactive effect

## Abstract

This study aims to explore the factors that influence the well-being situation of doctoral students in education from a qualitative perspective and draws on the ecological systems theory as an overarching framework. A total of 18 doctoral students in education from 3 universities in Hong Kong were interviewed. In line with the ecological systems theory, individual influential factors may embed and interact with all layers of systems (i.e., the microsystem, the mesosystem, the exosystem, the macrosystem, and the chronosystem) surrounding the doctoral students that hinder or boost their well-being, respectively. These six main areas of concern were identified from a thematic analysis of participants’ responses. The study highlighted several salient influential factors of doctoral students’ well-being, such as coping strategies, social relations, and their living and cultural environment. An interactive effect among specific factors, such as the COVID-19 pandemic and social movements, was also identified. Findings provide theoretical insights and offer recommendations for improving doctoral students’ well-being.

## 1. Introduction

Doctoral students are essential for the sustainability of the higher education system and make substantial social and economic contributions to the academic community after graduation [1]. On the strength of this importance, their well-being is of increasing concern worldwide [2]. Doctoral students’ well-being is not only significantly related to how individuals experience their doctoral program [3] but is also an important element that shapes the well-being of academics throughout their careers [1]. The well-being of doctoral students in education is even more critical as they will become educators after graduation. Therefore, their well-being may affect not only themselves but also their students. A ‘mental health crisis’ for doctoral students appears as they have reported a prevalence of well-being problems six times higher than those of the general population [4]. For instance, Berry et al. collected data from 3352 doctoral students from UK institutions that showed that 59% of the students reported a history of mental health problems [5]. These trends resonate with previous literature from different contexts (e.g., [3,6,7]). Moreover, the COVID-19 pandemic has further exacerbated their well-being problems by hindering doctoral students’ study progress, disrupting their learning environments, and increasing overall stress levels [8]. These studies have alerted us to draw attention to doctoral students’ well-being.

Although well-being problems have generally been well-documented, the overall status of doctoral students’ well-being and the factors that influence it are relatively underexplored from a qualitative perspective. The importance of how different factors can hinder or boost doctoral students’ well-being has attracted attention (e.g., [9,10]). Previous research studies have highlighted that academic communities and higher education institutions (e.g., [7]) play a vital role in the well-being of doctoral students. The social networks surrounding doctoral students, such as supervisors (e.g., [11]), peers (e.g., [12]), family, and friends (e.g., [6]), also play a key role in doctoral students’ well-being. Furthermore, doctoral students’ well-being is influenced internally by individual factors such as self-beliefs (e.g., [13]), engagement (e.g., [14]), and resilience (e.g., [15]). However, many of these studies have used a quantitative approach, focusing on the linear relations between well-being and the key factors that influence it.

Bronfenbrenner’s ecological systems theory of human development offers an optimal framework to investigate doctoral students’ well-being from a more holistic perspective [16]. This theory argues that individuals are influenced by multiple layers of the environment. Hence, this study draws on the ecological systems theory as the overarching framework to understand the status of doctoral students’ well-being, as well as the salient environmental factors that influence it. This study was conducted within the context of Hong Kong.

## 2. Theoretical Framework and Literature Review

### 2.1. Ecological Systems Theory

**Figure 1:**
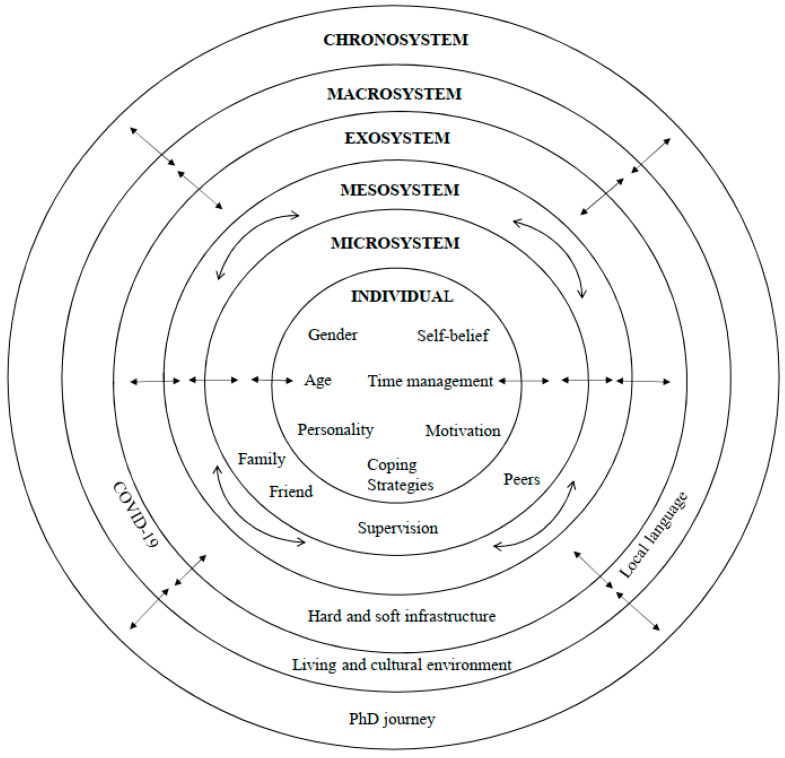
The ecological system model of PhD students.

Bronfenbrenner’s ecological systems theory of human development argues that the developing individual is influencing, and being influenced by, the environment at different levels [16]. This system comprises five socially organized subsystems (i.e., the microsystem, mesosystem, exosystem, macrosystem, and chronosystem) that help support and guide individual growth [17]. Although this theory was developed in the context of human development, it has also been successfully applied in the context of doctoral students’ well-being development (e.g., [9,18,19]). Ecological systems theory [17] proposes that individuals interact with five subsystems in their environment—the microsystem, mesosystem, exosystem, macrosystem, and chronosystem—which can also impact the whole well-being development of doctoral students [9]. The microsystem [17] is a pattern of activities, social roles, and interpersonal relations experienced by the developing person in a given face-to-face setting with particular physical and social characteristics (e.g., doctoral students’ family, supervisor, peer group, and other immediate relationships). Bronfenbrenner argued that the mesosystem refers to the connections and processes taking place between two or more microsystems surrounding the developing person (e.g., the relations between academic contexts and the home environment or work and life) [17]. The exosystem involves the linkage between a context in which an individual does not have an active role and the immediate context (e.g., the educational infrastructure of the doctoral students’ institution). The macrosystem consists of the broader culture, including the attitudes, social context, and prevailing norms that permeate the other systems (e.g., COVID-19, cultural environment, and societal norms). Bronfenbrenner commented that the chronosystem consists of change or consistency over time in an individual’s characteristics and the environment in which the people live (e.g., the different study stages during the doctoral journey) [17]. The framework is presented in Figure 1 below.

### 2.2. Doctoral Students’ Well-Being

Well-being is a broad construct [20]. Medin and Alexanderson described well-being as “the individual’s experience of his or her health” [21] (p. 75). This experience is not simply the absence of a mental illness, such as depression or anxiety, but rather the extent to which a person is emotionally, psychologically, and socially well [22]. Although many scholars have defined well-being, this comprehensive view of well-being has also been adopted by doctoral education researchers to highlight individuals’ constantly changing experiences [20]. When considering the state of doctoral students’ well-being, most of the studies have focused on well-being problems, such as anxiety, suicidal thoughts (e.g., [3]), depression (e.g., [23,24]), and burnout (e.g., [25,26]). However, well-being is not just about these negative states. Hence, it is important to have a more holistic view of doctoral students’ well-being [27]. While the mental health challenges faced by doctoral students have been widely documented across disciplines, the experiences of doctoral students in education may require special attention. Therefore, this study particularly aimed to investigate the status of overall well-being (i.e., emotional, psychological, and social) [28] among doctoral students in education. Specifically, emotional well-being refers to the experience of pleasant emotions [29] and a cognitive appraisal of satisfaction with doctoral studies and life [28]. The psychological well-being of doctoral students emphasizes functioning in individual life [29]. Social well-being manifested more public and social standards through which doctor students evaluate their functioning in academic and community life [30]. Moreover, these three well-being components align with the World Health Organization’s (WHO) definition of “a state of well-being in which the individual realizes his or her own abilities, can cope with the normal stresses of life, can work productively and fruitfully, and is able to make a contribution to his or her community” [31] (p. 12). This three-dimensional well-being model [28] has also been widely utilized to explore and understand student well-being, e.g., [32,33].

### 2.3. Influential Factors of Doctoral Students’ Well-Being

Studies have generally focused on identifying influential individual and environmental drivers of doctoral students’ well-being. Based on the ecological systems theory, the influential factors of doctoral students’ well-being will be discussed from six perspectives: individual, microsystem, mesosystem, exosystem, macrosystem, and chronosystem.

#### 2.3.1. Individual Factors

The individual influential factors impinge on doctoral students’ well-being can be divided into unmalleable and malleable domains. The most common unmalleable factors are probably gender and age. Gender differences in the well-being of doctoral students have been discussed in different contexts. For instance, Corvino et al. found that female doctoral students have lower well-being scores than men in the Italian context [34]. However, Joseph found that gender and well-being were not significantly associated with each other, but age was positively related to well-being in India [35].

The malleable factors include time management, financial pressures, self-belief, motivation, personality, and coping strategies. First, Beasy et al. identified that competing demands for time led to anxiety and ill-being [18]. Moreover, time pressures and lack of time to relax were related to doctoral student stress (e.g., [36,37]). Second, stress related to financial impact was also relevant as it increased an individual’s uncertainty and time pressure [36]. Third, the majority of studies investigated the relations between students’ self-beliefs and well-being [27]. Fourth, the majority of doctoral studies found that work engagement, intrinsic motives, or self-motivation were not only positively associated with students’ well-being but also with their academic outputs (e.g., [12,14]). Fifth, some individualized characteristics may hinder (e.g., perfectionism) or boost (e.g., emotional stability) the development of students’ well-being [9]. Finally, coping strategies such as planning, self-care management (e.g., [9]), emotional intelligence, and resilience [15] were regarded as important ways to manage the demands of doctoral study and protect well-being.

#### 2.3.2. Microsystem

The microsystem provides important support networks surrounding doctoral students. It includes relationships with one’s supervisors, peers, family, and friends [9]. There has been much discussion of the impact of problems from the supervisory relationship on doctoral students’ well-being [38]. In addition, scholars investigated the value of peer support in doctoral students’ well-being. For example, peers’ supporting roles positively affect well-being [39]. Students also described the value of support from peers and identifying with other doctoral students [27]. Furthermore, support from family and friends can also contribute to doctoral students’ well-being [40].

#### 2.3.3. Mesosystem

This system captures perceptions of the interrelationships and intersections between two or more microsystems, such as the relations between academic contexts and the home environment [9], or work and life, while work-life imbalance is the most important reason for psychological distress [41]. Moreover, Jackman, Sanderson et al. identified that students with childcare responsibilities found it difficult to secure sufficient time to work on their research [9].

#### 2.3.4. Exosystem

The exosystem, comprised of the hard infrastructure and soft infrastructure from the doctoral students’ institution context, is perceived to impact well-being. Indeed, Caesens et al. found that perceived institutional support has a direct positive impact on job satisfaction and a direct negative impact on perceived stress and sleep problems [12]. As for hard infrastructures, the facilities and workspaces (e.g., software, libraries, and physical spaces) were found to be important for students’ well-being [9,42]. Further, the soft infrastructures, including writing group programs [43], funding support, university policies and regulations, and other institutional structures [9], were integral for helping students face the challenges and stress associated with the doctoral journey. Therefore, the institutional infrastructure positively influences doctoral students’ educational experiences and improves their overall well-being.

#### 2.3.5. Macrosystem

The macrosystem consists of the wider culture, including the social context, attitudes, and prevailing norms (e.g., the COVID-19 pandemic, academic context) that permeate the other systems [8]. Moreover, oppressive research cultures characterized by long working hours, a competitive environment, and high levels of publication pressure undermined doctoral students’ mental health and well-being (e.g., [9]).

#### 2.3.6. Chronosystem

The chronosystem consists of change or consistency over time in the characteristics of an individual and of the environment in which the people live [17] (i.e., the different study stages during the doctoral journey). Examining the existent literature, previous studies have only considered the influence of the chronosystem by focusing on either the whole doctoral journey or the first year of doctoral study as the specific educational stages in the context (e.g., [9,18]). However, this may ignore the system impact on, and interaction with, individuals and the other four subsystems. Thus, this study aimed to situate the influential factors in all five subsystems and explore individual drivers and their interactive effect under the ecological systems framework of doctoral students. This study has three core research questions:

RQ1: What are the perceptions of the current status of doctoral students’ well-being?

RQ2: What are the most salient factors at the six layers that affect doctoral students’ well-being?

RQ3. How do these factors across the six layers interact to affect doctoral students’ well-being?

## 3. Method

### 3.1. Participants

A total of 18 doctoral students in education from 3 publicly funded universities in Hong Kong were recruited. These 18 students were interviewed individually. There were approximately 66.7% (*N* = 12) females and 33.3% (*N* = 6) males. Around 80% are aged 25 to 30 (*M* = 28.9 years). The ratios of participants who studied from Year 3 occupied the maximum proportion with 44.4%, followed by Year 1 (27.8%) and Year 2 (27.8%).

### 3.2. Interview Protocol

The development of the interview protocol is guided by the ecological systems model. Based on the insights gained from the literature review and the theoretical framework, the protocol is formulated with two main parts. The first part addresses RQ1, which aims to assess the students’ emotional, social, and psychological well-being. Each question is designed to elicit participants’ responses, encouraging them to express their feelings and self-perceptions. The second part addresses RQ2 and RQ3, focusing on identifying the influential and interactional factors that may impact doctoral student well-being. Questions are designed to explore individual, microsystem, mesosystem, exosystem, macrosystem, and chronosystem influences, ensuring a holistic approach to understanding their well-being. The initial draft of the interview questions was shared with academic peers and advisors for feedback. Based on the feedback received, the final version of the interview protocol was established. During the interview procedure, the interviewers summarized the key points that participants had shared in order to check whether they had correctly grasped and interpreted all the expressed opinions. Each interview was conducted for around 1 h, and all interviews were audio recorded and verbatim transcribed.

### 3.3. Data Procedure and Analysis

Thematic analysis was used to analyze the interview data. Thematic analysis [44] is a theoretically flexible method focusing on patterned meaning identified across a qualitative dataset. Based on the three research questions, three steps of thematic analysis were utilized to analyze the interview data [44]. In Step 1, the participant’s status of well-being was coded according to the well-being scale (a continuum from 1 to 10) of the three kinds of well-being (RQ1). In Step 2, the team coded the influential factors according to all five subsystems of the ecological systems framework (RQ2). In Step 3, the interactive effect of different factors was coded (RQ3). Please see Table S1 for coding examples.

## 4. Results

### 4.1. The State of Doctoral Students’ Well-Being

All the participants were invited to rate their current well-being status from zero (no sense of well-being) to ten (highest level of well-being) and share their perceptions. The outcome showed an average well-being score of 6.80 (*SD* = 1.44). Of the three perspectives of well-being, the score of emotional well-being ranked highest (*M* = 6.98, *SD* = 1.42, followed by psychological well-being (*M* = 6.95, *SD* = 1.83) and social well-being (*M* = 6.75, *SD* = 2.28). The details of well-being status will be discussed below from positive and negative perspectives.

#### 4.1.1. Emotional Well-Being

Regarding emotional well-being, the participants’ responses were clearly divided into high and low levels, while the vast majority (14 out of 18, with 77.8%) reported higher levels of emotional well-being. The participants who gave a higher rating for emotional well-being mentioned happiness, satisfaction, and interests in life. As one participant shared:

*I am now in a state of life with a higher level of well-being. I feel satisfied and happy. Although some things are not done well, there is no better solution in the short term. I think I have relatively high well-being right now and I would give a seven (for well-being), a score above the middle.*

*[U2-G2-S2]*

Generally, doctoral students in education who have a higher state of emotional well-being reported more positive emotions, such as *“I feel I love life, and I am optimistic all the time” [U2-G1-S1]*. Although they encounter some unpleasant situations in life or study, they can find other ways to adjust themselves dialectically in time, such as *“I am not really clear about my further research direction, which reduces my well-being, but I will adjust it through more communication with my classmates.” [U2-G2-S2].* On the other hand, those who reported a lower level of emotional well-being reflected more negative feelings of fatigue, stress, and exhaustion, such as *“I feel depressed and powerless” [U1-G1-S2].*

#### 4.1.2. Social Well-Being

The statements describing participants’ state of social well-being were divided into three parts, that is, their sense of belonging to the research team, the university, and the broader Hong Kong society. Compared to belonging to the university and society, most doctoral students (11 out of 18, with 61.1%) in education expressed they have high well-being within their research group through great interaction and mutual support. Well-being can be reflected in both the learning process and daily routine. As explained by a student:

*I think I have strong belonging in my experimental group. We often discuss with our supervisor and keep in touch all the time. Our group members will eat together, and organize activities together such as mountain climbing.*

*[U1-G2-S2]*

#### 4.1.3. Psychological Well-Being

For psychological well-being, most respondents (13 out of 18, 72.2%) reported a positive attitude on the whole, although a small number with two respondents gave a low score to their levels of psychological well-being. Most of them were able to make a positive self-assessment of their mental health and performed a combination of feeling good and functioning effectively. As one student shared:

*I think my mental state is relatively stable, and I found my efforts and return are proportional in the current phase, which makes me feel happy.*

*[U3-G1-S1]*

In conclusion, the participants generally reported high levels of emotional well-being, but a few expressed lower emotional well-being due to academic stress. For social well-being, participants showed higher belonging to their research team and university but lower well-being toward Hong Kong society. Moreover, most of the participants enjoyed high levels of psychological well-being, having positive attitudes toward their mentality and study, while a very small number had lower psychological well-being due to being troubled by mental illness symptoms.

### 4.2. Influential Factors of Doctoral Students’ Well-Being

The influential factors were classified into the six layers (i.e., individual factors and the five layers of surrounding environments) of the ecological systems theory. The themes were coded from positive and negative perspectives.

#### 4.2.1. Individual Factors

Individual factors can be divided into two perspectives in this study, namely unmalleable and malleable. For the unmalleable ones, age and gender were mentioned as first-layer influential factors. The following quotations supported the effect of age and gender:

*I am younger compared with my peers so I have more time to improve my skills, which makes me feel calm and optimistic.*

*[U1-G1-S2]*

Malleable personal factors were identified as personality, coping strategies, motivation, and self-beliefs. Firstly, participants who were extroverts and had positive characters reported higher well-being:

*I am an optimistic person, which makes me positive toward different things. Although I can’t stay positive for a long time, I find it easier to get satisfaction and happiness compared with my peers.*

*[U2-G1-S1]*

Second, participants mentioned that various coping strategies effectively impact their well-being. For example, participants who have higher resilience and do regular sport exercise reported higher well-being:

*I am not really clear about my further research direction, which reduces my well-being. However, I adjust it through more communication with my classmates. Participating in social activities eliminates my occasional boring feelings about the study.*

*[U2-G2-S2].*

Third, many interview responses also expressed that their well-being was affected by both internal and extrinsic motivation. On the one hand, better intrinsic motivation gives participants positive well-being. For example, one participant said:

*I am really interested in the topic of my doctoral dissertation as it was decided by myself. I feel more motivated to implement my project. It gives me a sense of accomplishment when I reach each small goal.*

*[U1-G1-S2]*

Taken together, in addition to unmalleable factors such as gender and age, participants’ personality, coping strategies, motivation, and self-belief as malleable factors are predictors of well-being enhancement from the individual perspective.

#### 4.2.2. Microsystem

The relationship between participants and their families, peers, and supervisors can affect their well-being from a microsystem aspect. The encouragement and support from family members were the solid backing for doctoral students in education to keep moving forward. As one participant shared:

*The most important thing is the support from your family. No matter where you are, you will be more peaceful, more stable, and have better resilience if you have inner support from your family.*

*[U1-G1-S3]*

Moreover, different relationships between participants and their supervisors significantly affected their well-being. Healthy and harmonious relationships showed that supervisors are good at encouraging and supporting doctoral students. They provided doctoral students with clear guidelines and moderate freedom, which led to a higher level of participants’ mental health. The following statements support this view:

*My supervisor is so nice and will give you clear and logical instructions. Working with him makes you believe your study goals can be achieved, that’s why I feel less worried compared with other doctoral students. *

*[U2-G2-S5]*

#### 4.2.3. Mesosystem

The relations between two or more microsystems are considered a mesosystem. In this study, lots of participants reported two or more factors from family and school occurring at the same time that affected their well-being in a negative way:

*Besides being a doctoral student, I also play a role as a mother, so I need to balance my family and study. I worry about how I can spend my time with my family member and my peers in school in a proper way so as to maintain a good relationship with them.*

*[U2-G2-S3]*

The related responses from the mesosystem showed that the tendency of participants’ anxiety increased when they needed to consider various interpersonal relationships under different circumstances and the interrelations between two or more settings they participated in. But a few said the mesosystem had positively influenced them, for example, *“Because I am married and I have a stable marriage relationship. I don’t need to take too much care of my family so that I can pay more attention to my own studies” [U3-G2-S1].*

#### 4.2.4. Exosystem

Participants also mentioned various resources from the university via the exosystem aspect as being influential factors. These resources consist of support from both hard and soft infrastructures.

In terms of hard infrastructure, most of the participants agreed that their university provided good conditions. As one participant shared:

*I like the office environment and the access to electronic resources and online journal articles provided by the university.*

*[U1-G2-S1]*

Soft infrastructure mainly provided positive effects on participants’ well-being. Participants from all the universities shared that their schools had provided and arranged counseling, workshops, or other activities to care for doctoral students’ mental health and relieve their anxiety. Below is an example:

*I participated in an online group arranged by my university. We shared our recent psychological status and suffering, and then the professional instructor would give us some suggestions to adjust our mentality.*

*[U3-G2-S3]*

#### 4.2.5. Macrosystem

According to the participants, the macrosystem included COVID-19, language, and living environment. Most doctoral students in education stated that COVID-19 had a significant negative impact on their well-being, both on study and daily life. The severe circumstances of the pandemic prompted all these government-funded universities to change their teaching mode from face-to-face to online, which significantly affected the study experience of doctoral students in education. One participant conveyed:

*We attended classes and seminars face to face before, which gave us plenty of time to communicate with professors and our classmates. But now we are just limited to online meetings. Sometimes doctoral students would suddenly go offline during the process. These experiences influence my well-being.*

*[U1-G1-S2]*

The living conditions and cultural environment were also mentioned by the participants. Convenient transportation, abundant recreational activities, and preferential treatment for doctoral students made participants have more chances to experience diversity and enhance their identity in Hong Kong. One doctoral student shared his thoughts on it:

*The preferable treatment of doctoral students in Hong Kong is obvious. Tickets for many entertainment activities can be half price, and there is student discount for MTR, which makes me feel very welcomed. In these aspects, I think the well-being of living and studying in Hong Kong is very high.*

*[U1-G1-S3]*

#### 4.2.6. Chronosystem

The whole doctoral journey was also mentioned as a significant factor that affected participants’ well-being. Participants who just start the study journey normally experience a high level of life satisfaction:

*I have just started my doctoral study, so I don’t feel too much pressure right now. I am very interested in scientific research and I feel quite fresh toward research and life, without feeling very bored and burnt out.*

*[U1-G1-S3]*

### 4.3. Interactive Effects Influence Doctoral Students’ Well-Being

According to the interviews, participants reported that influential factors interactively affect their well-being, especially under the circumstances of COVID-19 and the social movements of Hong Kong. These were organized based on the six layers of the ecological systems theory.

Some participants mentioned that the impact of COVID-19 was multifaceted. It significantly influenced one’s research progress, maintenance of relationships, utilization of infrastructure, and living conditions. The following extract supported it:

*COVID-19 really affects our well-being in various ways. The most obvious change is that it makes it difficult to collect data due to the policy restrictions, which definitely delayed my research process. I also could not return to my hometown to see my parents. Moreover, the social distancing measures reduced opportunities to interact with my peers which was the most common way to release my pressure. Even the chance to decompress by using the university’s sports facilities is gone.*

*[U3-G2-S2]*

As for social movements, the 2019 Hong Kong protests disrupted the social order seriously. The normal teaching activities of the university have been damaged, and participants’ well-being has been noticeably affected from different perspectives. The interactive factors focus mainly on personal coping strategies, relationships with peers, and support from the university. As one participant conveyed:

*The conflict of consciousness brought by social movements helped me learn to deal with problems more rationally, and I managed my emotions well when I received some unfriendly treatment. When I was out, I felt scared as I could not speak the local language. Of course, our university and my supervisor also pay special attention to our mental health and personal safety during this period. However, it is inevitable that the conversations between some of my peers with different perspectives became more cautious, which did have some impact on our relationship.*

*[U3-G1-S3]*

In conclusion, the factors that affect doctoral students’ well-being can not only be derived from one specific system but also can interact with multiple aspects, such as the personal, microsystem, and exosystem, at the same time.

## 5. Discussion

The findings of this study highlight the unique well-being needs of doctoral students in education. Despite the positive and negative aspects of doctoral student well-being that have been investigated, a more nuanced picture of the factors that hinder and boost the well-being of doctoral students, especially those in the education field, needs further discussion. A particular interest has been laid in the influential factors of doctoral students’ well-being. In line with the ecological systems theory, the individual factors were also embedded into all five systems (i.e., microsystem, mesosystem, exosystem, macrosystem, and chronosystem) surrounding doctoral students in education.

First, from the perspective of individual factors, the findings of the study indicated that both of the unmalleable factors (i.e., gender and age) were related to student well-being. For instance, female doctoral students may perceive more pressure than male students, which is consistent with previous studies [34]. This may be because people often accept that in addition to the role of doctoral students, females are expected to take up family responsibilities and childcare. In line with this circumstance, females are generally more emotional compared with males, so they could be affected more deeply after experiencing adversity or stress [45]. Moreover, the study indicated that the younger students feel calm and optimistic, while the older students were more likely to feel distressed, which is consistent with previous studies [35].

In addition, the study identified that the malleable factors of individuals, including coping strategies, motivation, and self-belief, were associated with doctoral students’ well-being. The study found that students who have coping strategies such as higher resilience and regular sport exercise reported higher levels of well-being. This is echoed by the finding that resilience played a vital role in managing the demands of doctoral study and boosting doctoral students’ well-being [15]. Moreover, this study is consistent with the finding that exercise was regarded as the positive factor that related to the mental health and well-being of doctoral students [46]. As expected, both intrinsic and extrinsic motivation help doctoral students maintain a good state of well-being, which resonates with previous studies (e.g., [46]). However, the study indicated the dual influence of self-belief on well-being. Doctoral students with a lack of confidence feel negative towards life, which has a great impact on their mood. This is consistent with the findings of numerous studies (e.g., [34]). On the other hand, students with higher self-efficacy were more likely to feel happiness and accomplishment, which is consistent with previous studies (e.g., [41]).

Interestingly, time management does not seem to be that important for the doctoral students’ well-being, which is inconsistent with previous studies (e.g., [18,47]). They found that the most highly cited word in the literature was ‘time’, which also resonated with the literature [41]. However, this study showed that time management was rarely reported by students as an influential factor. This may be because the stress related to financial impact was interwoven with time pressure, and financial pressure seemed more acute [36]. However, these pressure factors may be affected by the financial resources provided by higher educational institutions in the Hong Kong context. Therefore, it still needs further longitudinal investigations.

Second, the microsystem in this study consisted of family and friend relations, peer relations, and supervision relations, which are important networks influencing doctoral students’ well-being. As expected, this study found that students with more family support reported higher status of well-being, which echoes with previous studies (e.g., [6]). However, the dual influence of peer and supervision relations was emphasized in this study. For instance, positive-going communication between peers and being approved by them, alleviating the pressures and workloads brought about by competitive cultures, was identified as an effective way to improve doctoral students’ well-being, which resonates with previous studies (e.g., [27,39]). In contrast, some doctoral students also expressed they struggled if they had bad interpersonal interactions with their peers. This may be because of the competitive environment in the Hong Kong academic context.

This study also indicated that a supervisor can either have positive or negative effects on doctoral students’ well-being, which is consistent with past studies (e.g., [36,38]). Supervisors who provided students with clear guidelines and moderate freedom led to a higher level of well-being among doctoral students in education. However, students’ well-being would be greatly thwarted if their supervisors tended to wear them down with heavy workloads. This also resonated with the finding [9] that supervisors who defied what doctoral students perceived as a broader culture of long working hours in academia helped to encourage self-care (individual) and management of the work-life intersection (mesosystem). Thus, there is clearly a need to explore problems of supervision further in the context of the whole research ‘ecosystem’ [48].

Third, the mesosystem captured perceptions of the interrelationships between two microsystems such as family and university in this study. The results indicated that doctoral students in education with multiple family roles or family problems were more likely to suffer from study pressure, which really affected their well-being status. This is consistent with the findings of previous studies [9]. However, students with stable and positive marriage relationships can pay more attention to their studies, which protects their general well-being. This is echoed by the finding that positive life-work relations particularly buffer exhaustion (e.g., [25]).

Fourth, the exosystem includes hard and soft infrastructure in this study. In the matter of hard infrastructure, most of the participants identified that universities provided sufficient facilities to better support the well-being of doctoral students, which is agreed with by numerous studies (e.g., [9,42]). Moreover, students with limited support, such as those who do not have office space, were more likely to feel inconvenienced and exhausted. However, the results indicated that soft infrastructure including scholarships, workshops, and other health-related training programs mainly provided positive effects on participants’ well-being, which is consistent with numerous studies (e.g., [40,43]).

Fifth, the macrosystem, which consists of the COVID-19 pandemic, living conditions, cultural environment, and the local language in Hong Kong, had a significant impact on doctoral students’ well-being. As expected, the epidemic prompted all universities to change their teaching and learning modes, which significantly affected the study experience and thwarted the well-being status. The quarantine policy of COVID-19 not only created anxiety but made students suffer from a lack of companionship with family and friends. These results are consistent with previous studies (e.g., [8,49]., 2021). Moreover, the specific language environment of Hong Kong obviously restrains the living convenience and well-being of doctoral students who cannot speak Cantonese. This is echoed by the finding that doctoral students with a lower level of English language proficiency in the UK have a lower probability of well-being satisfaction [50]. However, the results indicated a contradictory effect of the living conditions and cultural environment on doctoral students’ well-being. Interestingly, one of the students has indicated receiving preferable treatment in Hong Kong. This could be attributed to the scholarship funding provided by the University Grants Committee for PhD students in Hong Kong [51] and the preferential treatment in public transportation for younger students, creating a relatively unique advantage.

Finally, in the chronosystem, the whole doctoral journey was regarded as a significant factor that affected participants’ well-being. The results indicated that doctoral students in education reported higher levels of life satisfaction during the early stage of the doctoral journey. However, the doctoral students in the later stage of the study experienced more stress and depression. This may be consistent with the finding that doctoral students report the highest well-being during the coursework phase, while the dissertation phase was found to be the most challenging for most students, as indicated by the lowest wellness scores [52]. Hence, under the ecological systems, most of the influential factors indicated dual effects that both strengthen and restrain the doctoral students’ well-being.

These influential factors not only have significant influences on well-being, but their interactive effects also influence doctoral students’ well-being. This study outlines two specific factors (i.e., COVID-19 and the social movements) in the Hong Kong context. The results highlighted the interactive and domino effect of COVID-19 (macrosystem) on doctoral students’ research process (individual), the maintenance of relationships (microsystem), and the utilization of infrastructure (exosystem). This, in turn, influences students’ mental state and inhibits students’ well-being, which is consistent with the effect of COVID-19 in previous studies (e.g., [8,49]). Interestingly, this study found some positive interactive effects of COVID-19. The results indicated that doctoral students sense of resilience was enhanced (individual), they adapted to the online learning system (exosystem), and their meeting effectiveness with supervisors and peers (microsystem) was reinforced during the COVID-19 pandemic (macrosystem), which in turn strengthened their fulfillment and well-being. In addition, in the matter of social movements, doctoral students in education identified that their conscience aroused by the movements (macrosystem) boosted their sense of rationality and their ability for emotional regulation (individual). However, there is limited research investigating the positive and negative interactive effects of factors (e.g., the COVID-19 and the social movement) on doctoral students’ well-being.

## 6. Implications

Supporting the well-being of doctoral students in education has far-reaching implications for future development of education, considering that these students are not only the next generation of education scholars, but also the future leaders, policymakers, and changemakers who will shape the education landscape. This study has conceptual, theoretical, and practical implications. First, the study investigated the influential factors that boost and hinder doctoral students’ well-being, which contributes to the conceptual understanding of what protective factors can promote doctoral students’ well-being in their doctoral study. In line with the ecological systems theory, this study identified some interactive effects of the factors from the perspectives of individuals and all layers of subsystems on doctoral students’ well-being. Second, this study investigated the individual influential factors, including coping strategies and time management, contributing to the conceptual understanding of how different factors affect doctoral students’ well-being. This can help provide doctoral students in education who experience poor well-being with the right tools to help themselves acquire more appropriate skills or strategies (e.g., exercise habits, time management) to enhance their well-being (e.g., [36,53]). Third, supervisors play a crucial role (e.g., esteem support, emotional support, and being responsive and accessible) in the doctoral project progress and students’ well-being, and hence, influence whether the student may drop out of the program or not [40]. Supervisors should improve their knowledge, effectiveness, and competence to protect students’ well-being [11], while policymakers or the institutions could provide effective learning communities, workshops, and other resources to support supervisors’ protective implementations. Fourth, to adequately address doctoral students’ well-being problems, the institutions and policymakers may have to develop prevention and early intervention strategies and policies to promote well-being in doctoral students, such as providing sufficient accommodation and language (i.e., Cantonese) training programs to doctoral students in the Hong Kong context.

## 7. Limitations and Directions for Future Research

Despite the contributions of this study, there are also several limitations. First, the participants volunteered to take part, so the findings could be susceptible to self-selection bias. Future studies could adopt multiple evaluation methods (e.g., observation and supervisor reports) to explore more authentic results from various angles. Second, the self-reported interviews in this study were collected at a single time point. Future studies may use longitudinal or experimental studies to explore relationships among them. Third, this study examined the roles of individual and environmental factors drawn from the ecological model. However, the reality is likely more complex, and this study might have failed to uncover other pertinent factors. Future studies may try to use mixed-methods approaches or more authentic approaches (i.e., observation, well-being detection) to understand the comprehensive and interactive effects of more systems in the ecological systems theory.

## Data Availability

Data will be made available upon request.

## Supplementary Materials

The following supporting information can be downloaded at: https://www.mdpi.com/article/10.3390/bs14100929/s1, Table S1. The coding examples.
