# Supplementary material for: The Well-Being of Doctoral Students in Education: An Ecological Systems Perspective
Source: Behav Sci (Basel). 2024 Oct 10;14(10):929. doi: 10.3390/bs14100929 (PMC11505550; doi:10.3390/bs14100929)
Supplement: Supplementary file 1 [file behavsci-14-00929-s001.zip › behavsci-3195657-supplementary.pdf]

Supplementary Materials

Table S1. The coding examples.

No	Theme	Sub-Theme	Category	Quotation	Remarks
1	State of Well-being	Emotional WB	Positive	I feel satisfied and happy.	U2-G2-S2
			Negative	I feel depressed and powerless.	U1-G1-S2
		Social WB	Positive	I think I have strong belonging in my experimental group.	U1-G2-S2
			Negative	I have never thought I am a part of Hong Kong society in my mind.	U1-G1-S1
		Psychological WB	Positive	I think my mental state is relatively stable and I found my efforts and return are proportional in the current phase.	U3-G1-S1
			Negative	I usually suffer from insomnia, which really affects my psychological health and self-efficacy.	U3-G2-S1
2	Influential factors	Individual	Positive	I am an optimistic person, which makes me positive toward different things.	U2-G1-S1
			Negative	Gender stereotypes affect my emotions during the study.	U3-G2-S3
		Microsystem	Positive	I felt relaxed when I communicated with my supervisor. He encourages us to discover research topics by ourselves, combing what we are interested in and what we are good at.	U3-G1-S3
			Negative	I felt anxious when I found other peers had finished many tasks one day and faster than me.	U2-G2-S2
		Mesosystem	Positive	I have a stable marriage relationship. I don't need to take too much care of my family so that I can pay more attention to my own studies.	U3-G2-S1
			Negative	I worry about how I can spend my time with my family member and my peers in school in a proper way so that to maintain a good relationship with them.	U2-G2-S3
		Exosystem	Positive	The scholarship provided by the school made we can meet our daily needs and reduce our economic burden.	U3-G1-S2
			Negative	I didn't have an office at first, which made me very down.	U2-G1-S1
		Macrosystem	Positive	Tickers for many entertainment activities can be half price, and there are student discounts for MTR, which makes me feel very friendly.	U1-G1-S3
			Negative	I can't speak Cantonese and sometimes I get discriminated against because of it.	U3-G1-S1
		Chronosystem	Positive	I have just started my PhD study, so I don't feel too much pressure right now.	U1-G1-S3
			Negative	I strongly feel the pressure to promote my research project in Year 3 now.	U1-G1-S2
3	Interactive effect	Positive		Online learning actually allowed me to schedule studies more flexibly and save commuting time. Also, zoom meetings with my supervisor and peers sometime would be more effective.	U2-G2-S4
		Negative		I became afraid of socializing and prefer to stay alone, which meant I didn't take full advantage of the resource and related services offered by my university to some extent.	U1-G2-S3
